# Light- and magnetically actuated FePt microswimmers

**DOI:** 10.1140/epje/s10189-021-00074-1

**Published:** 2021-06-02

**Authors:** Vincent Mauricio Kadiri, Jan-Philipp Günther, Sai Nikhilesh Kottapalli, Rahul Goyal, Florian Peter, Mariana Alarcón-Correa, Kwanghyo Son, Hannah-Noa Barad, Michael Börsch, Peer Fischer

**Affiliations:** 1 Max Planck Institute for Intelligent Systems, Heisenbergstr. 3, 70569 Stuttgart, Germany; 2 Institute of Physical Chemistry, University of Stuttgart, Pfaffenwaldring 55, 70569 Stuttgart, Germany; 3 Jena University Hospital, Friedrich-Schiller University Jena, Nonnenplan 4, 07743 Jena, Germany

## Abstract

Externally controlled microswimmers offer prospects for transport in biological research and medical applications. This requires biocompatibility of the swimmers and the possibility to tailor their propulsion mechanisms to the respective low Reynolds number environment. Here, we incorporate low amounts of the biocompatible alloy of iron and platinum (FePt) in its $\hbox {L1}_{{0}}$ phase in microstructures by a versatile one-step physical vapor deposition process. We show that the hard magnetic properties of $\hbox {L1}_{{0}}$ FePt are beneficial for the propulsion of helical micropropellers with rotating magnetic fields. Finally, we find that the FePt coatings are catalytically active and also make for Janus microswimmers that can be light-actuated and magnetically guided.

**Supplementary Information:**

The online version supplementary material available at 10.1140/epje/s10189-021-00074-1.

## Introduction

Biomedical applications of micro- and nanoparticulate carriers based on liposomes and viruses or virus-like particles mostly rely on passive diffusion to reach their target site [1, 2]. Such systemic applications require larger doses and are generally wasteful as most carriers do not reach their intended target [2]. It is therefore of interest to ask whether biocompatible actively propelled micro- or nanocarriers can enable targeted delivery.

**Fig. 1:**
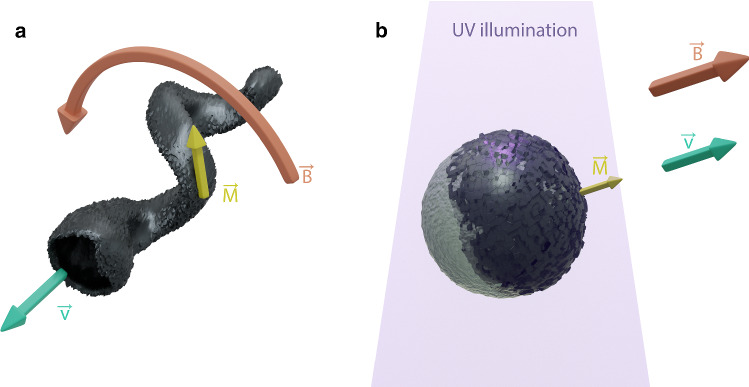
$\hbox {L1}_{{0}}$ FePt is a versatile hard magnetic material that can be nanostructured to yield **a** helices propelled by a rotating magnetic field ([12] and this work) and **b** light-actuated Janus particles propelled by self-phoresis and directed by a static magnetic field (this work). The propulsion velocity $\overrightarrow{v}$ is pictured in green, remanent magnetization $\overrightarrow{M}$ is shown in yellow, applied (rotating) magnetic field $\overrightarrow{B}$ in orange and UV illumination in purple

If synthetic analogues are to propel like swimming microorganisms, they have to do so under the constraints of low Reynolds number hydrodynamics ($\hbox {Re}<1$), where the viscous forces dominate over inertial forces [3]. Active swimming in Newtonian fluids, like water, thus requires propulsion mechanisms based on non-reciprocal motion to overcome the scallop theorem [3]. An exception is only provided in non-Newtonian viscoelastic fluids, where even a reciprocal motion can lead to propulsion [4]. While it is challenging to develop force-free microswimmers that propel by metachronal body shape changes [5], it is also possible to mimic the flagellar motion of bacteria by exerting a torque on helical micro- and nanopropellers with the help of an external rotating magnetic field (see Fig. 1a) [6, 7]. These helical structures have also been successfully propelled through biological fluids by decreasing their size in order to pass through the macromolecular mesh of a hyaluronan gel [8], by coating them with enzymes such that they can liquefy mucines [9] or by coating them with non-adhesive perfluorocarbons so that they can be moved through the vitreous body of the eye [10]. In all these cases, the propellers require a hard ferromagnetic section that can be used to propel the structures by rotation–translation coupling.

The alloying of platinum and iron can yield such a hard magnet, which in addition is non-toxic, unlike many other magnetic materials [11, 12]. FePt must be in the $\hbox {L1}_{{0}}$ phase to exhibit these favorable magnetic properties. As such, $\hbox {L1}_{{0}}$ FePt has garnered significant attention as a non-cytotoxic, rare earth-free, hard magnetic material with some of the highest energy products observed to date [13, 14]. As we demonstrated recently, $\hbox {L1}_{{0}}$ FePt microhelices are not cytotoxic and are thus suitable for biomedical applications [12, 14]. The possibility to form nanostructures of $\hbox {L1}_{{0}}$ FePt via the co-deposition of iron and platinum in a physical vapor deposition chamber at glancing angles opens further avenues for the application in microdevices, two of which are explored here. First, we show that reducing the FePt coating thickness can improve the magnetic properties. In addition, we show that the FePt coating is also catalytically active. This opens the possibility to realize light-actuated iron–platinum microswimmers that are propelled by self-phoresis (see Fig. 1b) [15].

## Methods

### Fabrication of $\hbox {L1}_{{0}}$ FePt Janus particles and helices

FePt-coated helical microswimmers were fabricated on a monolayer of $1~\upmu \hbox {m}$ polystyrene beads. 1 mL polystyrene beads (2.5 wt%, Kisker Biotech) suspended in water were centrifuged at 14 000 rcf for 5 minutes, and the supernatant was removed and then re-dispersed in deionized water. This cleaning step was repeated and an equal volume of ethanol was added. The particles were assembled at the air/water interface of a 0.1 mM sodium dodecyl sulfate (SDS) solution. The dispersion was slowly pipetted onto a silicon slide partially submerged at a $45^{\circ }$ angle in the liquid phase, to create a particle monolayer at the air–liquid interface. The resulting monolayer was transferred onto a fresh silicon wafer via an automated Langmuir–Blodgett system [16]. After drying, the substrate was exposed to an oxygen plasma (200 W, 0.7 mbar) for 15 minutes to reduce the size of the beads to 500–600 nm. Onto these seed particles, $1.5~\upmu \hbox {m }\hbox {SiO}_{{2}}$ helices were grown via glancing angle deposition (GLAD) at a deposition angle of $83^{\circ }$ [16, 17]. A 5 nm adhesion layer of Ti was grown at $18^{\circ }$ before Fe and Pt were co-deposited at a 50:50 atomic ratio at a deposition angle of $18^{\circ }$ while rotating the substrate. The FePt layer was grown with thicknesses between 50 nm and 200 nm. Finally, the wafer was annealed in a vacuum ampoule at $680~^{\circ } \hbox {C}$ for 1 h.

Janus particles were fabricated in a similar way to those fabricated with $\hbox {Pt-SiO}_{{2}}$ [18, 19]. Briefly, $1~\upmu \hbox {m}$ $\hbox {SiO}_{{2}}$ beads were transferred onto a silicon wafer via Langmuir–Blodgett deposition on which a 5 nm Ti adhesion layer was first grown to minimize the lattice mismatch between $\hbox {SiO}_{{2}}$ and FePt. A thin film of 50:50 co-deposited Fe and Pt was then evaporated and the wafer was subsequently annealed in a vacuum ampoule at $680~^{\circ } \hbox {C}$ for 1 h to yield $\hbox {L1}_{{0}}$ FePt-coated Janus particles.

**Table 1:** Comparison of magnetic properties of $\hbox {L1}_{{0}}$ FePt helices for different fabrication schemes, including the optimized method of this paper

Nominal deposition thickness	Deposition angle $\alpha$	Remanence $M_{\mathrm {R}} \frac{{10}^{-6}\mathrm{{emu}}}{{\mathrm{{mm}}}^{2}}$	Saturation $M_{\mathrm {S}}$ $\frac{{10}^{-6}\mathrm{{emu}}}{{\mathrm{{mm}}}^{2}}$	Remanence $M_{\mathrm {R}}$ $\frac{{10}^{-12}\mathrm{{emu}}}{\mathrm{{helix}}}$	Saturation $M_{\mathrm {S}}$ $\frac{{10}^{-12}\mathrm{{emu}}}{\mathrm{{helix}}}$
500 nm [12]	$83^{\circ }$	11.2	12.2	2.4	2.6
100 nm	$45^{\circ }$	$22.9\pm 0.2$	$34.3\pm 0.9$	19.8	29.7
200 nm	$18^{\circ }$	$65.9\pm 0.2$	$101.2\pm 0.4$	57.1	87.6
100 nm	$18^{\circ }$	$37.0\pm 5.0$	$52.0\pm 1.0$	32	45
50 nm	$18^{\circ }$	$18.9\pm 0.5$	$27\pm 7$	16.4	23.4

Scanning electron micrographs were acquired on a Zeiss Gemini scanning electron microscope (SEM).

Energy-dispersive X-ray photoelectron spectroscopy (EDX) scans were performed on a Zeiss SESAM transmission electron microscope at 200 kV with an EDX Noran system and Pathfinder 1.1 software.

### SQUID magnetometry

The magnetic properties of wafer pieces with FePt helices (annealed) and Janus particles (as-deposited, annealed, and aged) were characterized via superconducting quantum interference device (SQUID, MPMS-7, quantum design) magnetometry. The field-dependent magnetization measurements were conducted at room temperature and the field was varied from 7 T to $-7$ T. For the *M*–H hysteresis loop measurements, the external magnetic field H has been applied in the out-of-plane and the in-plane directions where in-plane denotes a measurement perpendicular to the wafer surface’s normal while out-of-plane is defined as parallel to it. The contribution of the substrate and the $\hbox {SiO}_{{2}}$ was subtracted via linear fitting at high field ranges.

### Oxygen evolution

Wafer pieces $(\sim 2~\hbox {cm}^{{2}})$ of annealed and as-deposited FePt Janus particles were submerged in a 10% (w/v) $\hbox {H}_{{2}} \hbox {O}_{{2}}$ solution inside a round-bottomed flask. The gas evolution was quantified with and without UV light with a measuring cylinder over time via displacement of the water by the $\hbox {O}_{{2}}$ gas. UV illumination was controlled via a UV LED (M365LP1—365 nm, 2000 mW, 1700 mA, ThorLabs).

### Particle tracking

Janus particles were removed from the wafer using sonication and then dispersed in solutions with $\hbox {H}_{{2}} \hbox {O}_{{2}}$ concentrations of 0, 2 and 5% (w/v). The particles were imaged with a Zeiss Axiovert microscope using a 40 X objective with and without UV light $(320\hbox { mW cm}^{{-2}})$. Images were acquired at 15 fps for 60 seconds and particle diffusion was evaluated via a custom python script based on TrackPy [20, 21], and MSD data fitted to obtain the effective diffusion coefficients *D* [22].

### Magnetic actuation

The magnetic helices were magnetized in-plane with a 2 T electromagnet while they were still attached to the wafer. Then the magnetized helices were sonicated to yield final concentrations of $10^{{5}}$ particles/mL and actuated as described in previous publications [6, 10]. A frequency sweep using rotating magnetic fields with strengths of 2 and 4 mT was performed and imaged on a Zeiss Axiovert microscope using a 63 X objective. The magnetic field was applied via a custom-built 3-axis Helmholtz coil [16].

Similarly, Janus particles were magnetized out-of-plane and magnetically guided using the same setup with a static, homogeneous magnetic field of 3.5 mT.

### ARPE-19 cell culture

ARPE-19 cells were cultured in Dulbecco’s modified Eagle medium (DMEM 1 g/L Glucose, Gibco) with 10% (w/v) fetal bovine serum (FBS, Gibco) and 1% (w/v) Penicillin-Streptomycin. Cells were incubated in an atmosphere of 5% $\hbox {CO}_{{2}}$ at $37~^{\circ } \hbox {C}$ and passaged every 7 days at a ratio of 1:7.

### Cell viability

Cell viability in the presence of FePt microswimmers was analyzed via a modified standard protocol of “LIVE/DEAD$^\mathrm{TM}$ Fixable Red Dead Cell Stain Kit, for 488 nm excitation” (Invitrogen). ARPE-19 cells were incubated with a 100:1 ratio of particles to cells. After incubation for 24 or 72 h, LIVE/DEAD$^\mathrm{TM}$ Fixable Red Dead Cell Stain was added in a ratio of $1 \,\upmu \hbox {L}$ for every $\sim 10^{{6}}$ cells and incubated for 30 min. The medium was then aspirated and the cells washed with PBS, fixed with a 3.7% (w/v) paraformaldehyde solution and washed with PBS three more times. The cells were permeabilized with 0.1% (w/v) Triton-X solution. Immunostaining was then performed with DAPI ($4^\prime$,6-diamidino-2-phenylindole, Invitrogen). The cells were subsequently imaged on a Zeiss LSM 900 Airyscan microscope with a 20 X objective, and fluorescence data were extracted and evaluated via a custom-written CellProfiler pipeline  [23].

**Fig. 2:**
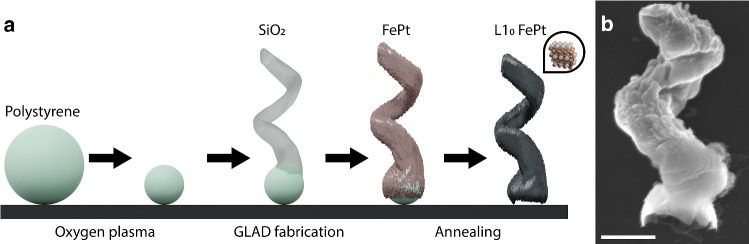
**a** Optimized fabrication scheme to generate, **b** helices (scanning electron micrograph) with higher magnetizations. The scale bar is 500 nm

### XPS

X-ray photoelectron spectroscopy (XPS) measurements were taken on the sample using a Theta Probe Angle-Resolved X-ray Photoelectron Spectrometer System (Thermo Fisher Scientific Inc.). For the excitation, a non-monochromatic Al K$\alpha$ source was used, which was kept at 100W (h$\upnu =1486.68$ eV). The survey scan was acquired with an energy pass of 200 eV, followed by high-resolution spectra acquisition of the various elements using an energy pass of 50 eV. The quantitative analysis was executed using Avantage software; at first, a nonlinear Shirley background was removed from the high-resolution core-level spectra of the elements; then, the peak analysis and fitting were done using the Powell method. The charge referencing was corrected by adjusting the C–C (or C–H) component of the C 1s peak to 284.8 eV.

**Fig. 3:**
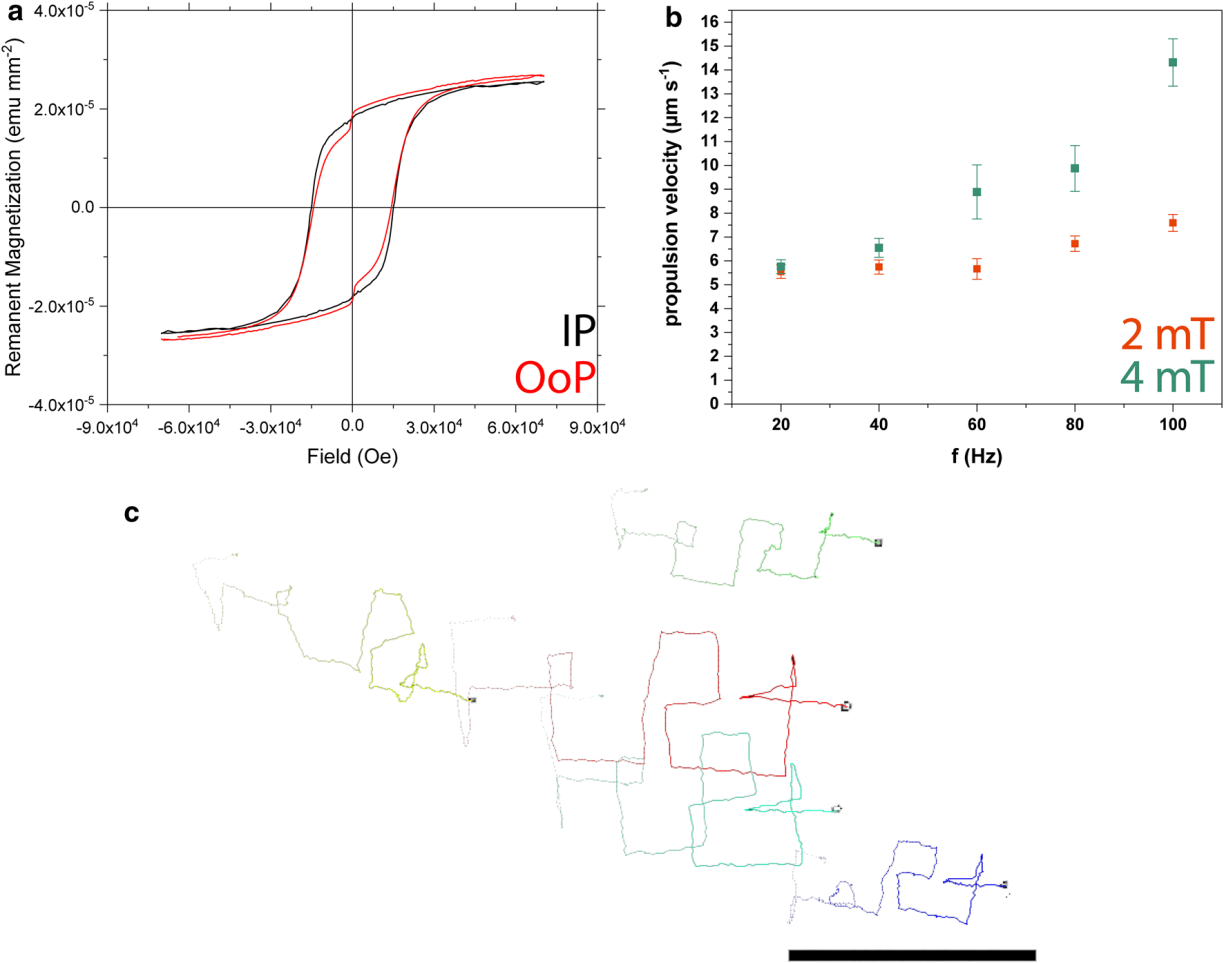
**a** Magnetic hysteresis curve for optimized helical microswimmers (50 nm, $18^{\circ }$). **b** Propulsion velocities for frequency sweeps with a magnetic field of 2 (orange) and 4 mT (green), respectively. **c** Optimized helical microswimmers spelling out “FePt.” Scale bar is $50 \,\upmu \hbox {m}$

**Fig. 4:**
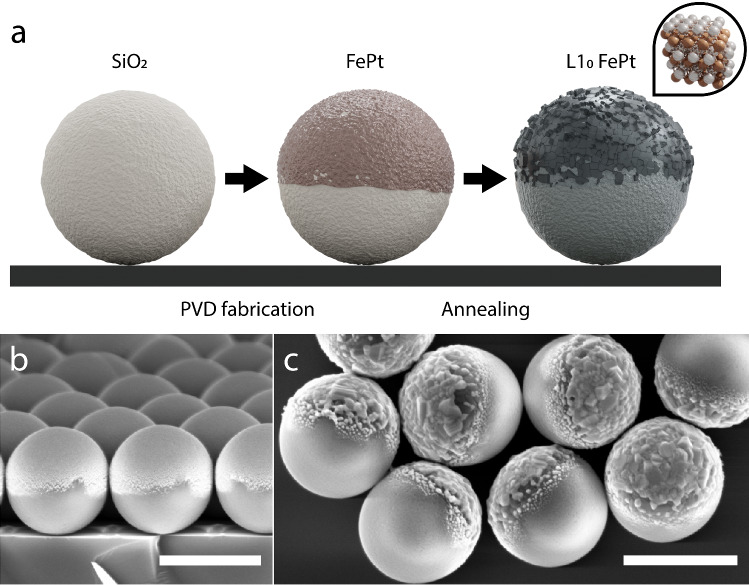
**a** Fabrication of photocatalytic $\hbox {L1}_{{0}}$ FePt Janus particles and scanning electron micrographs, **b** before and **c** after annealing at $680~^{\circ } \hbox {C}$ and sonication. Scale bars are $1~\upmu \hbox {m}$

## Results and discussion

### Magnetically driven FePt microswimmers

We first show that the magnetic properties of helical microswimmers have been optimized by an improved fabrication method. In contrast to the direct deposition of Fe and Pt onto silica helices [12], the optimized FePt helices of this work require one-tenth of the material during deposition while enabling an eightfold increase in remanent magnetization per helix (see Table 1). Two effects may explain this significant increase in magnetic performance: firstly, a new deposition scheme, which is schematically shown in Fig. 2a, and secondly, the reduction in the thickness of the FePt coating. Previously, FePt helices were fabricated by depositing $\hbox {SiO}_{{2}}$ helices onto a 500 nm $\hbox {SiO}_{{2}}$ bead seed layer on which FePt, at a nominal thickness of 500 nm and an angle of $83^{\circ }$, was then grown. In this work, we use 500-600 nm polystyrene beads (after oxygen plasma etching), which are spaced $\sim 1~\upmu \hbox {m}$ apart, as seeds (see Experimental section and Fig. 2a). After growing the $1.5~\upmu \hbox {m }\hbox {SiO}_{{2}}$ helix, between 50 and 200 nm FePt is co-deposited and annealed (see Fig. 3). Crucially, the FePt is not deposited under oblique incidence, but at a reduced angle. Following Hawkeye et al. [17], this shadowing constraint follows from the deposition geometry:

1 $$\begin{aligned} h~\tan \alpha \le d-w, \end{aligned}$$

where *h* is the height of the growing particle, $\alpha$ the deposition angle, *d* the center-to-center distance of seed particles and *w* the width of the particle. In the present geometry, $h=1.5~\upmu \hbox {m}$, $w=0.5~\upmu \hbox {m}$, $d=1~\upmu \hbox {m}$, which yields a deposition angle of $\alpha =18^{\circ }$. This reduced angle allows for the incident vapor to penetrate in between the helices while avoiding inter-seed deposition onto the substrate.

As a result of coating the helices at $18^{\circ }$ instead of $83^{\circ }$, the entire helices are covered with a thin layer of FePt. While this means that it is more difficult to estimate the magnetization per volume (emu $\hbox {cm}^{{-3}})$, the magnetization per surface area (emu $\hbox {mm}^{{-2}})$ or per helix can still be determined accurately. It should be noted that because of the new deposition scheme, the amount of helices covering the wafer’s surface per $\hbox {mm}^{{2}}$ is reduced by a factor of 4 ($\sim 2.5$ x $10^{{9}}$ particles per 2 inch wafer) compared to samples that are fabricated on close-packed seed layers. Nevertheless, the samples grown at $18^{\circ }$ with a nominal FePt thickness of 50 nm still exhibit an almost twofold increase in magnetic remanence $(19~\upmu \hbox {emu}\, \hbox {mm}^{{-2}})$ compared with the helices that have previously been grown at $83^{\circ }$ with a nominal thickness 500 nm, which only showed $11.16~\upmu \hbox {emu}~\hbox {mm}^{{-2}}$. In other words, changing the deposition scheme optimizes material use by a factor of 20 and a factor of 80 when evaluating on a per helix basis. Beside this, the magnetic coating’s curved geometry may also have a favorable effect on the magnetization [24]. Table 1 shows that the deposition of material up to a thickness of 200 nm at $18^{\circ }$ leads to an increase in remanence. Depositing more than 200 nm would lead to the fusion of helices and was thus not investigated. We find that depositing at higher angles leads to more material loss and lower magnetization, while the increase in remanence levels off. The latter may be explained by the critical domain size for FePt. Above the critical domain size, $\hbox {L1}_{{0}}$ FePt nanomagnets no longer possess a single domain structure. Kikuchi et al. observed a critical domain size of 50 nm for 10 nm FePt layers [25]. One would thus expect that while the saturation magnetization increases beyond thicknesses of 50 nm, the remanence will level off, as is observed in Table 1 (and [12]). The helices in this work (see Fig. 2b) are coated with a thin $\hbox {L1}_{{0}}$ FePt film that covers the entire helix, as compared to helices with a 350 nm magnetic section that were used previously and that showed a much smaller remanence (see Table 1 and [12]).

As the hysteresis curve in Fig. 3a illustrates, the FePt helices retain their hard magnetic behavior despite the reduction in the thickness of the FePt coating compared to previous work.

**Fig. 5:**
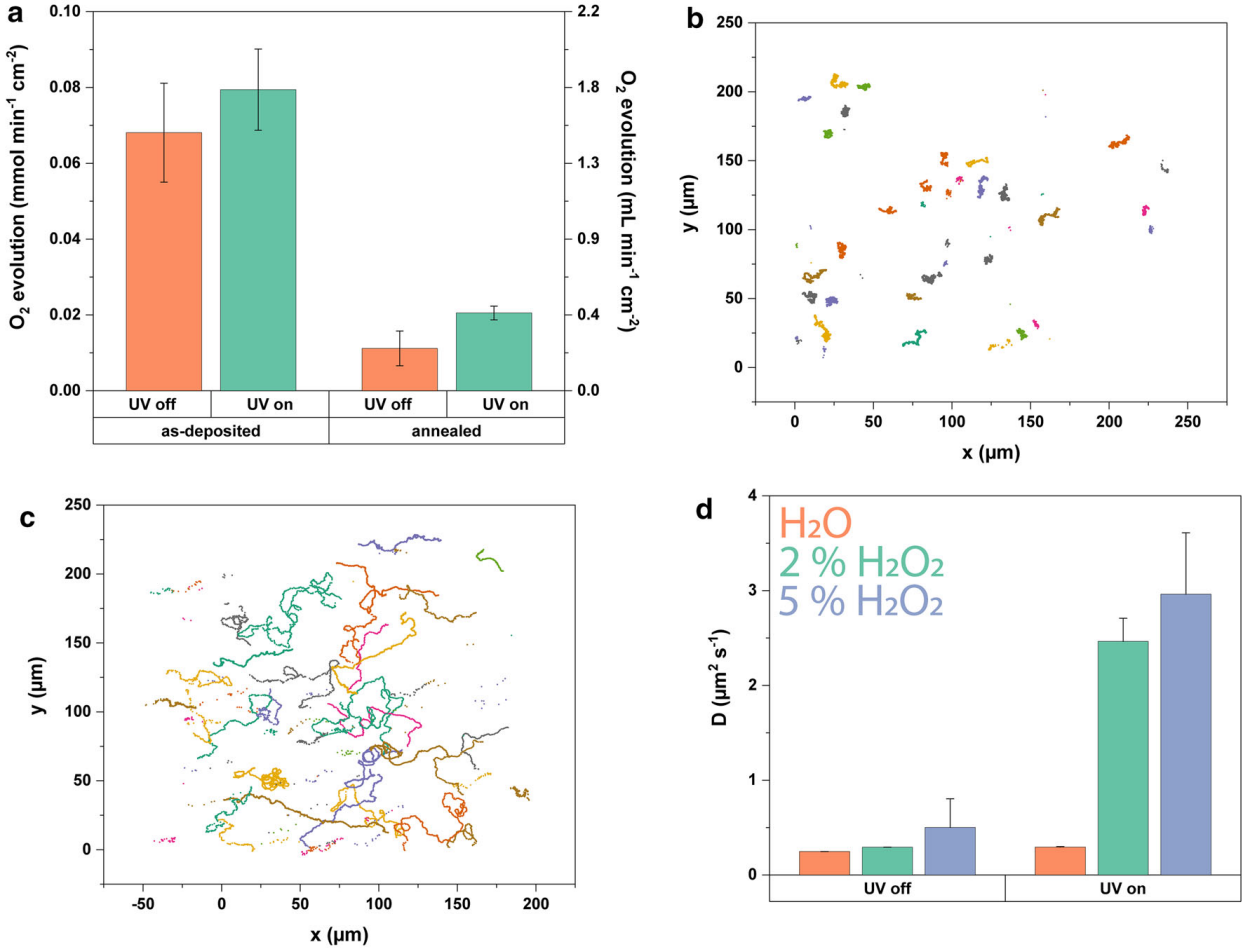
**a** FePt-coated wafers catalyze the decomposition of hydrogen peroxide. In the case of annealed $\hbox {L1}_{{0}}$ FePt, UV irradiation leads to a twofold increase in activity. Particle tracking data confirms this effect for FePt-Ti- coated Janus particles in 2% (w/v) $\hbox {H}_{{2}} \hbox {O}_{{2}}$ solutions, which diffuse slower **b** without UV compared to **c** those with UV irradiation. **d** Effective diffusion coefficients obtained from particle tracking show a significant diffusion enhancement upon UV illumination in $\hbox {H}_{{2}} \hbox {O}_{{2}}$

Depositing a thinner layer of FePt also did not lead to a significant decrease in propulsion speeds, as shown in Fig. 3b for rotating external fields of 2 and 4 mT. In contrast to previous FePt helical microswimmers [12], we did not detect a step-out frequency below 100 Hz for either field strength. We associate this observation with the higher magnetic moment [26]. Similarly, the maneuverability was unaffected (Fig. 3c and Supplementary Video 1). Thus, optimized $\hbox {L1}_{{0}}$ FePt helices conserve material without sacrificing the magnetic performance of these actuated microstructures.

### Catalytic microswimmers based on FePt

Apart from the magnetic properties, FePt nanoparticles have previously shown catalytic activity in heterogeneous Fenton-like reactions [27]. Moreover, both iron oxides and platinum are commonly used (green) catalysts [18, 27–29], and thus, it is interesting to examine whether the FePt-coated structures also display catalytic activity. This would permit the realization of chemically propelled self-phoretic microswimmers that could also be magnetically guided by virtue of the $\hbox {L1}_{{0}}$ FePt. The versatility of the FePt nanostructuring allows for the facile fabrication of Janus particles based on $\hbox {SiO}_{{2}}$ with a 5 nm Ti adhesion layer as shown in Fig. 4a–c. The as-fabricated particles were immersed in a solution containing 10% (w/v) $\hbox {H}_{{2}} \hbox {O}_{{2}}$ and the oxygen gas was quantified with the aid of a measuring cylinder [18].

The as-deposited 50 nm film of FePt was initially found to catalyze $\hbox {H}_{{2}} \hbox {O}_{{2}}$ decomposition at a rate of $0.07\pm 0.01$ mmol ($=1.5$ mL) per min and $\hbox {cm}^{{2}}$ of wafer (see Fig. 5a). For reference, Choudhury et al. [18] previously reported a Pt film of 20 nm nominal thickness catalyzing the same reaction at a rate of 1 mmol ($=22.4$ mL) $\hbox {min}^{{-1}}\,\hbox {cm}^{{-2}}$. Thus, the co-deposition of Pt with Fe certainly leads to a decrease in reactivity. Annealing the wafer to obtain $\hbox {L1}_{{0}}$ FePt led to a further drop in activity. Nevertheless, a small activity of 0.01 mmol ($=0.2$ mL) $\hbox {min}^{{-1}}$ $\hbox {cm}^{{-2}}$ of $\hbox {O}_{{2}}$ was still measured.

At first glance, it would thus seem that the excellent magnetic and the catalytic properties of FePt are mutually exclusive. Surprisingly, we did find, however, that irradiation with UV light led to a photo-switchable statistically significant twofold increase in oxygen evolution (Fig. 5a). Over the course of multiple experiments, a permanent increase of activity after UV illumination was also observed. The UV light does not lead to a significant increase of oxygen evolution on the as-deposited FePt. Thus, the annealing step, which is also crucial for generating the as-prepared $\hbox {L1}_{{0}}$ phase FePt coating, seems important for this photocatalytic effect, too.

After sonicating the particles off the wafer, they were dispersed in 2 and 5% (w/v) $\hbox {H}_{{2}} \hbox {O}_{{2}}$ solutions to examine if $\hbox {L1}_{{0}}$ FePt–Ti–$\hbox {SiO}_{{2}}$-based Janus particles self-propel. Supplementary Videos 2 and 3, respectively, depict the Janus particles without and with UV illumination at 2 % H2O2. Tracking data of these videos is also displayed in Fig. 5b, c. Supplementary Videos 4 and 5 are at 5 % H2O2. Dispersing the particles in hydrogen peroxide solutions results in enhanced diffusion upon UV irradiation. As expected, the $\hbox {L1}_{{0}}$ FePt Janus particles dispersed in water showed no significant increase in their diffusion coefficient upon UV irradiation (Fig. 5d).

**Fig. 6:**
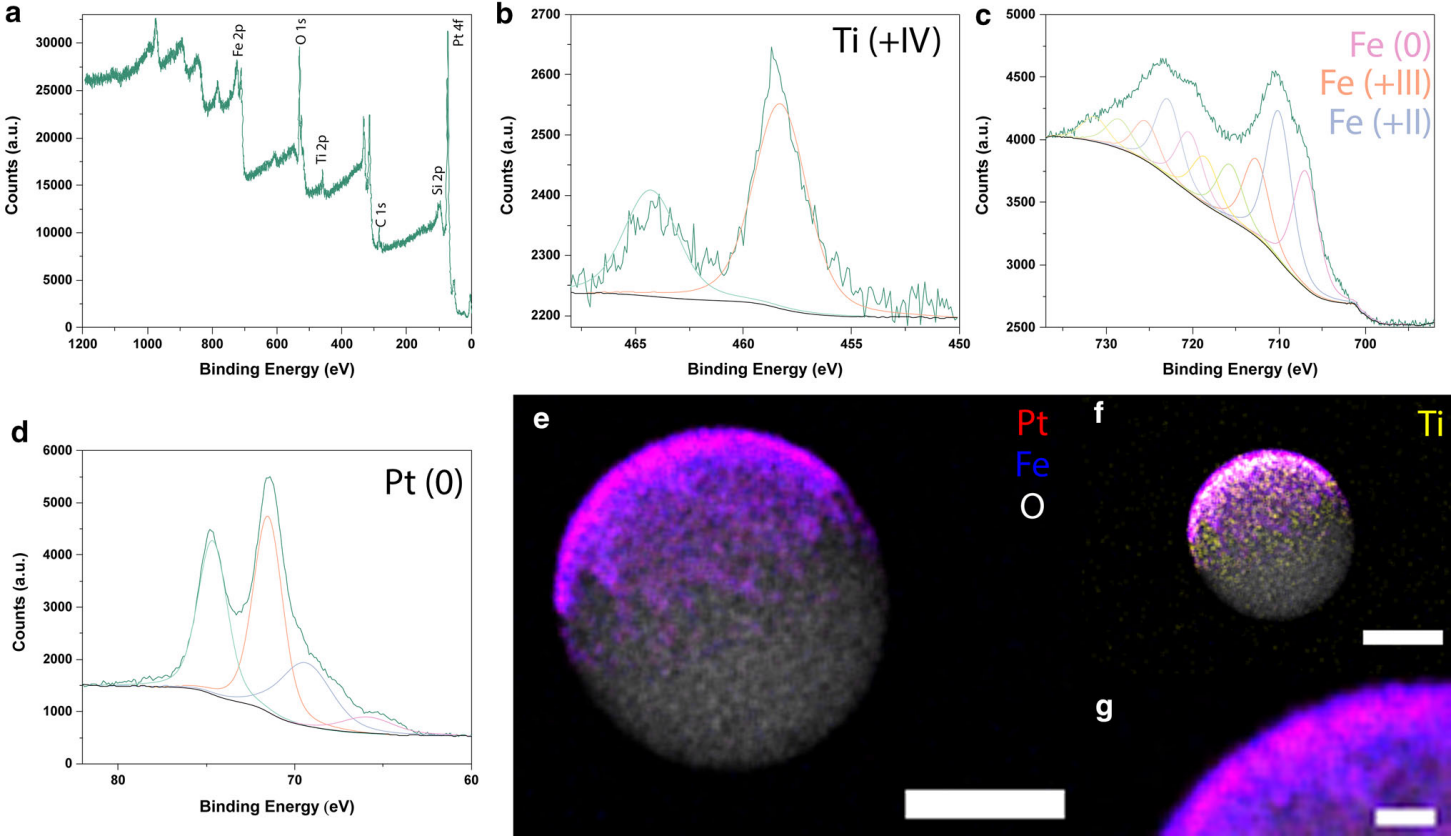
**a** Surface characterization via X-ray photoelectron spectroscopy (XPS) yielded information on oxidation states of elements present in $\hbox {L1}_{{0}}$ FePt Janus particles. In **b**, Titanium was exclusively present as Ti($+\hbox {IV}$), while in **c** the iron species were found to be present in the oxidation states (0), ($+\hbox {II}$) and ($+\hbox {III}$), suggesting the presence of both metallic iron and iron oxides. **d** Platinum was not oxidized. **e** Energy-dispersive X-ray spectroscopy (EDX) imaging in a transmission electron microscope (TEM) showing the location of Fe (blue), Pt (red), O (white) and **f** Ti (yellow) on the Janus particles. Scale bars are 500 nm. **g** Distribution of Fe around the outer regions of the FePt layer. Scale bar is 50 nm

X-ray photoelectron spectroscopy (XPS) was conducted to characterize the $\hbox {L1}_{{0}}$ FePt-based Janus particles as well as shed light on a possible mechanism underlying the photocatalytic properties. Iron, oxygen, titanium, carbon, silicon and platinum peaks were all readily identified (Fig. 6a), and the overall Fe-to-Pt surface ratio was determined to be 52:48. Titanium, which was added as a 5 nm adhesion layer between the silicon dioxide particles and the FePt layer, was found at an atomic fraction of 9.7% when compared to FePt (Fig. 6b). Titanium was exclusively found with an oxidation state of ($+\hbox {IV}$) suggesting oxidation via ambient oxygen either during annealing or from the underlying silicon dioxide (probably forming $\hbox {TiO}_{{2}})$. While platinum was solely present in the oxidation state (0), iron peaks for Fe(0), Fe($+\hbox {III}$) and Fe($+\hbox {II}$) were identified and fitted (Fig. 6c, d), thereby confirming that the deposited iron must have similarly reacted with oxygen during annealing.

FePt nanoparticles form a thin ($\sim 2$ nm) $\hbox {FeO}_{\mathrm {x}}$ passivation layer upon annealing, as was recently reported [30]. The high-resolution XPS spectrum of the Fe 2p levels indicates the presence of Fe($+\hbox {III}$) typically seen in a $\hbox {Fe}_{{2}} \hbox {O}_{{3}}$ crystal structure [31]. Palacci et al. have shown that $\hbox {Fe}_{{2}} \hbox {O}_{{3}}$ is photocatalytically active and enables active motion of hematite-based Janus microswimmers, and hence, iron oxide may also underlie the FePt-based particles’ photocatalytic activity [28, 32].

However, our fabrication scheme (Fig. 4a) alsoincluded a 5 nm Ti adhesion layer, which turned to $\hbox {TiO}_{{2}}$ after annealing at $680~^{\circ } \hbox {C}$, i.e., above the annealing temperature for the anatase $\hbox {TiO}_{{2}}$ crystal structure [33]. Therefore, one cannot completely rule out that $\hbox {TiO}_{{2}}$ contributes to the photocatalytic properties, since anatase-$\hbox {TiO}_{{2}}$ is a well-known semiconductor and photocatalyst, with a band gap of 3.2 eV. The particles were analyzed via energy-dispersive X-ray spectroscopy (EDX) to better understand the distribution of the Pt and Fe (Fig. 6e), and Ti species (Fig. 6f) on the particles. From the EDX (and XPS) results, we assume that Ti is localized more toward the sides of the particles as well as the interface between FePt and silicon dioxide, while both Fe and Pt can be found on the outer surface, with a higher concentration of iron than platinum on the outer layer (Fig. 6g). Combined with the knowledge of the presence of the oxidized Fe ($+\hbox {II}$, $+\hbox {III}$) species at the surface, this could also explain the particle’s photocatalytic activity as previously described by Richard et al. [28] and Palacci et al. [32]. However, the exact mechanism still needs to be determined.

**Fig. 7:**
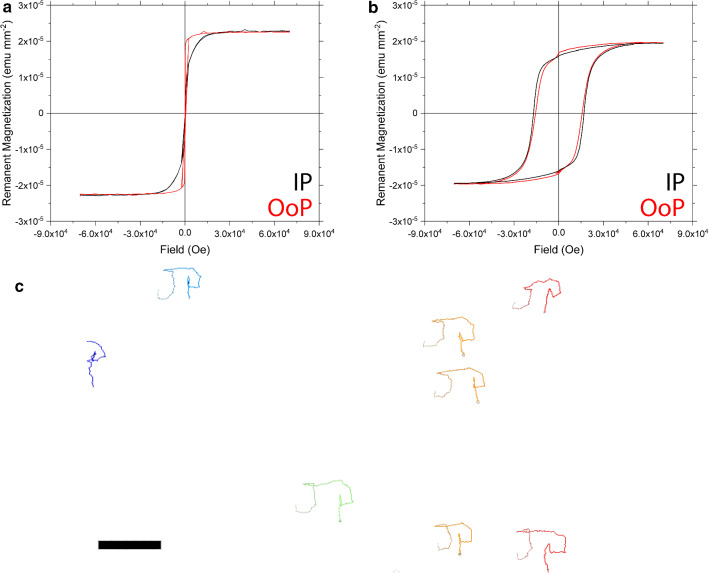
Magnetization hysteresis curve for FePt–Ti-based Janus particles **a** before and **b** after annealing. **c** Magnetically guided Janus particles spelling out “JP”

As SQUID characterization reveals, the as-deposited Janus particles exhibit a soft magnetic behavior and turn into hard magnets post-annealing (Fig. 7a, b). They can be magnetized out-of-plane, leaving them with a remanent magnetization on the order of $185 \hbox {emu cm}^{{-3}}$. In combination with the observed photo-switchable catalytic behavior, this yields light-activated magnetically steerable microswimmers. When the light is turned on and a static magnetic field is applied, the Janus swimmers will propel along the magnetic field lines (Supplementary Video 6, Fig. 7c). Similar properties were shown for sequentially deposited Co and $\hbox {TiO}_{{2}}$ [34, 35]. The ability to use $\hbox {L1}_{{0}}$ FePt, as discussed above, leads to more favorable magnetic properties, but it remains to be established to which extent the Ti and FePt layers, respectively, contribute to the microswimmers’ activity.

**Fig. 8:**
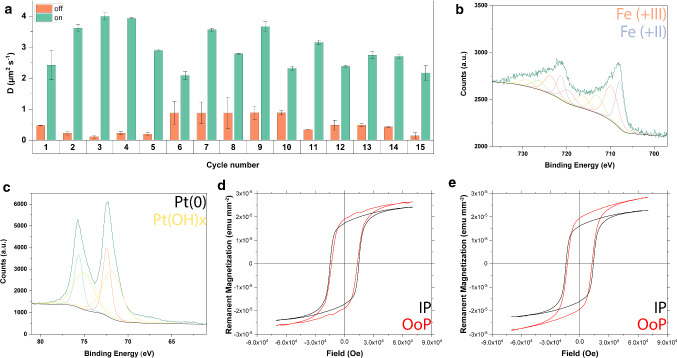
**a** FePt–Ti–$\hbox {SiO}_{{2}}$-based Janus particles exhibit photocatalytic behavior over the course of 15 cycles without any significant loss in switchability. **b** After aging in 10% (w/v) $\hbox {H}_{{2}} \hbox {O}_{{2}}$, the Fe(0) species observed in previously disappear while Fe($+\hbox {III}$) becomes more prevalent. **c** Pt(0) species, on the other hand become hydroxylated. The compositional changes on the surface do not, however, negatively affect $\hbox {FePt--Ti--SiO}_{{2}}$-based Janus particles’ magnetic properties. These stay hard magnetic **d** before and **e** after aging

We found that over the course of 15 UV off–on cycles, the photocatalytic activity was not impaired (Fig. 8a). The particles repeatedly stopped exhibiting enhanced diffusion upon switching the UV illumination off suggesting the particles do in fact exhibit switchable photocatalytic behavior.

Additional XPS studies revealed that after multiple decomposition cycles in 10% (w/v) $\hbox {H}_{{2}} \hbox {O}_{{2}}$, the Fe(0) shoulder observed in Fig. 6c disappears while Fe($+\hbox {III}$) species become more prevalent, while platinum becomes partially hydroxylated (Fig. 8b, c). Crucially, the overall magnetization of the particles remains unaffected (Fig. 8d, e). These results suggest that the involvement of Fe(0) at the particle surface is initially not catalytic since iron undergoes oxidation. Whether the Fe($+\hbox {II}$, $+\hbox {III}$) at the surface is involved in the catalysis after oxidation remains to be determined. Secondly, since oxidation does not negatively affect the overall hard magnetic properties, the oxidation step seems to be self-limiting, i.e., an iron oxide passivation layer protects the remaining $\hbox {L1}_{{0}}$ FePt from being oxidized. Thirdly, since the change of oxidation states of both iron and platinum on the surface (aging) does not seem to interfere with the swimmer’ switchable photocatalytic activity (Fig. 8a), it appears likely that the latter is due to the titanium adhesion layer.

To ascertain $\hbox {L1}_{{0}}$ FePt’s contribution toward the decomposition of hydrogen peroxide, we thus fabricated identical particles to those depicted in Figs. 4a–c and 6e–g without Ti. Oxygen evolution in 10% (w/w) $\hbox {H}_{{2}} \hbox {O}_{{2}}$ was low at first and comparable to non-aged annealed particles with titanium. Over the course of 45 minutes, the catalytic activity increased until the wafers eventually exhibited stable activities of $0.18\pm 0.01\hbox { mmol cm}^{{-2}}$ $\hbox {min}^{{-1}}$ post-aging (Supplementary Video 7). The activities for these wafers could not be switched via UV once this point was reached. We found that UV irradiation accelerates the aging process, which was also observed during the $\hbox {O}_{{2}}$ evolution experiments on the Janus particles containing Ti. We thus conclude that the fabricated structures contain $\hbox {L1}_{{0}}$ FePt in the core, but that iron oxide species and elemental platinum are also found at the surface and that these increase the catalytic activity. Their formation is accelerated by the use of UV light (e.g., a photo-accelerated catalyst).

The wafers age and over time become catalytically active irrespective whether they are illuminated with UV light or not. However, the FePt–Ti–$\hbox {SiO}_{{2}}$ Janus particles retain their photo-switchability over the time scale of the experiment (at least 40 min, with $\hbox {H}_{{2}} \hbox {O}_{{2}}$ concentrations of up to 5%), as shown in Fig. 8a. The aging thus does not seem to significantly affect the catalysis under these experimental conditions presumably due to the lower $\hbox {H}_{{2}} \hbox {O}_{{2}}$ concentrations and shorter time spans.

### Cell viability

**Fig. 9:**
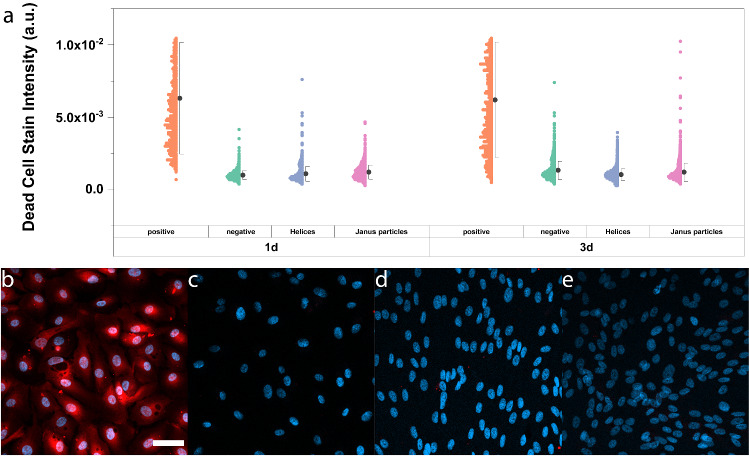
**a** Cell death as quantified by red fluorescent dead cell stain intensity after 1 and 3 days incubation for ARPE-19 cells with helical magnetic, Janus particle microswimmers, and positive (fixed cells) and negative controls ($100~\upmu \hbox {L}$ phosphate-buffered saline (PBS)), respectively. Corresponding fluorescence microscopy images after 3 days for **b** positive, **c** negative, **d** helices and **e** Janus particle samples. Cells were stained with DAPI (blue) and LIVE DEAD cell stain (red). Scale bar is $50 \,\upmu \hbox {m}$

Another very attractive property of $\hbox {L1}_{{0}}$ FePt is that it is not cytotoxic [12], such that FePt-based micro- and nanodevices are ideal for eventual application in vitro or in vivo biomedical settings. Since the details of the fabrication have been changed for these new structures, we also re-examined cell viability. We could show that the FePt microswimmers of this work do not exhibit any significant cytotoxicity as shown in Fig. 9a. For this, ARPE-19 cells (positive and negative sample Fig. 9b, c respectively) were incubated with helices at a 1:100 ratio and cell viability was unaffected by the presence of both helical (Fig. 9d) and Janus particle microswimmers (Fig. 9e) after 3 days.

## Conclusion

In this work, we investigated $\hbox {L1}_{{0}}$ FePt as a versatile platform for the construction of biocompatible microswimmers. As shown in earlier work, $\hbox {L1}_{{0}}$ FePt is a viable material for future application in biomedical micro- and nanodevices since it is not only non-cytotoxic, but also rare earth-free, while exhibiting hard magnetic properties after annealing. High coercivities in the range of $\sim 1.5~\hbox {T}$ make them hard to re-magnetize while their high magnetic remanence $(333\hbox { emu cm}^{{3}})$ allows for their actuation through biologically relevant fluids such as cell media via the application of relatively weak $\sim$1–4 mT magnetic fields.

We found that coating $\hbox {SiO}_{{2}}$ helices with 50 nm FePt thin films at lower incidence angles ($18^{\circ }$) led to an overall increase in magnetic remanence when compared to thicker 500 nm films grown at glancing angles. Magnetic performance and maneuverability of resulting microswimmers remained unaffected.

Depositing $\hbox {L1}_{{0}}$ FePt onto $1~\upmu \hbox {m}$ $\hbox {SiO}_{{2}}$ beads with a 5 nm Ti adhesion layer generated Janus particles, comparable to $\hbox {Pt-SiO}_{{2}}$-based Janus microswimmers. As-deposited FePt catalyzed the decomposition of $\hbox {H}_{{2}} \hbox {O}_{{2}}$ at a slower rate than pure platinum. The catalytic activity of FePt was further reduced after annealing. However, the activity increased upon UV irradiation. UV light can thus be used to realize photo-switchable, magnetically guided $\hbox {L1}_{{0}}$ FePt Janus particles. We found that titanium-free $\hbox {L1}_{{0}}$ FePt Janus particles exhibited an irreversible aging effect which over time restored post-annealing catalytic activities of up to $180 \,\upmu \hbox {mol cm}^{{-2}}$ $\hbox {min}^{{-1}}$. This effect has, to our knowledge, not been shown previously. Aging was photo-accelerated with UV even in the absence of titanium. Certainly, the use of hydrogen peroxide as a fuel is problematic for eventual application, replacing this fuel could therefore be subject of further investigations. However, chemically propelled microswimmers offer the possibility of realizing chemotaxis [36].

## Supplementary Information

Below is the link to the electronic supplementary material.

Supplementary material 1 (avi 11974 KB)

Supplementary material 2 (mp4 719 KB)

Supplementary material 3 (mp4 657 KB)

Supplementary material 4 (mp4 831 KB)

Supplementary material 5 (mp4 732 KB)

Supplementary material 6 (avi 4222 KB)

Supplementary material 7 (mov 10475 KB)

## Data Availability

This manuscript has data included as electronic supplementary material. [Authors’ comment: The online version of this article contains supplementary material, which is available to authorized users.]
